# To question whether we trust a learner is demoralising

**DOI:** 10.15694/mep.2017.000019

**Published:** 2017-02-01

**Authors:** Neel Sharma

**Affiliations:** 1 Albert Einstein College of Medicine

**Keywords:** Entrustment

## Abstract

This article was migrated. The article was marked as recommended.

## Letter

Entrustment is now the headliner in medical education. An addition to competency based medical education with a focus on whether we trust a learner or not to perform an activity unsupervised (Ten Cate O, 2013).

Whilst consensus has meant this concept has now taken a strong hold, I worry for my learners. Evidence suggests two things. Firstly trust by and large is not an issue that patients themselves are concerned with. They still recognise doctors as the most trustworthy professional (Skinner G, Clemence M, 2016). However it seems senior doctors feel otherwise. Secondly recent evidence from the UK at least has shown that the junior workforce is now more demoralised than ever (GMC, 2016). Whilst seniors may contest that in their day they were on site more frequently, we have to admit that the patient volume and complexity of cases has increased dramatically. Whilst maintaining an never ending service provision with less resources, learners are being bombarded with more and more barriers to progression. For seniors to question directly whether they trust a learner appears hostile at a time when we need to be more unified as a profession. We should as educators be aiming to support, value and motivate our learners who are already questioned and assessed no end; continuous qualifications and bursting portfolios of evidence is expected when we still recognise a focus on service provision as opposed to actual training (Millett D, 2016).

I am concerned with the movements being made in the field and the lack of understanding of the real life working issues learners are faced with. The next 50 years will see learner driver change on the issues that matter; lack of feedback, bullying and harassment in the work place and lack of preparedness.

## Notes On Contributors

Dr Neel Sharma has a keen interest in medical education and is currently based at the Albert Einstein College of Medicine, New York, USA

## Bibliography/References

Ten Cate O. Nuts and Bolts of Entrustable Professional Activities. J Grad Med Educ. 2013 Mar; 5(1): 157-158.

https://doi.org/10.4300/JGME-D-12-00380.1

Skinner G, Clemence M. Politicians are still trusted less than estate agents, journalists and bankers. 2016. Available from https://www.ipsos-mori.com/researchpublications/researcharchive/3685/Politicians-are-still-trusted-less-than-estate-agents-journalists-and-bankers.aspx

GMC. The state of medical education and practice in the UK report: 2016. Available from http://www.gmc-uk.org/publications/somep2016.asp

Millett D. Change medical training so all doctors can be generalists says GMC. 2016. Available from http://www.gponline.com/change-medical-training-so-doctors-generalists-says-gmc/article/1418971

## Declarations

The author has declared that there are no conflicts of interest.

