# Supplementary material for: To question whether we trust a learner is demoralising
Source: MedEdPublish (2016). 2017 Feb 1;6:19. [Version 1] doi: 10.15694/mep.2017.000019 (PMC10885221; doi:10.15694/mep.2017.000019)
Supplement: Peer review history

# Reviewer report: Reviewer response for version 1

**DOI:** 10.21956/mep.19435.r28593

**Published:** 2017-02-06

**Authors:** Peter Dieter

**Affiliations:** 1 AMSE

This review has been migrated. The reviewer awarded 4 stars out of 5 Trust is one of the most important things in our live, not only trust from the patient to the doctor, but also trust from the doctor to the patient, the teacher to the student, the student to the teacher, the employee to the employer, the employer to the employee and trust between partners and a married couple. In my experience from the last years trust disappears more and more in our society and distrust and fear is growing ever more. What the author describes in his letter is also a reflection on the change in our society and has to be changed. We all need to regain trust and not first to see other people with suspicion and fear. It will be difficult to do this in the medical field without a change within our society.

Reviewer Expertise:

NA

No decision status is available

# Reviewer report: Reviewer response for version 1

**DOI:** 10.21956/mep.19435.r28594

**Published:** 2017-02-04

**Authors:** Trudie Roberts

**Affiliations:** 1 Leeds Institute of Medical Education

This review has been migrated. The reviewer awarded 3 stars out of 5 An interesting letter which gave me pause to think but given the erudite discussion here I think I may have misinterpreted the issue. For me the term entrustment does not mean that I have a pejorative view of the individual as a person but simply that at this time in their development they may not be at a stage to undertake a certain procedure or action without support. Thus when my daughter was five years old I did not 'entrust' her to make a meal for herself using the cooker. When she was older and had the requisite experience not only did I entrust her but positively encourage this activity. Previously many trainees were asked to undertake actions they felt ill-prepared to undertake and I have many memories of this myself which still cause a shudder. Personally I would have like more support and supervision before being left isolated, alone and expected to cope. So this concept is a comment on the stage of development of the individual not a comment on their character. Perhaps we need to re-emphasise this to trainees.As an aside, the demoralisation of junior doctors in the UK has many origins but these mainly relate to being valued as a person, not being used as political pawns and supported in doing the job they thought they had signed up to do i.e. care for patients. Good training programmes with support and appropriate supervision from seniors who care about, and for them is the only thing keeping many of them working in the NHS.

Reviewer Expertise:

NA

No decision status is available

# Reviewer report: Reviewer response for version 1

**DOI:** 10.21956/mep.19435.r28592

**Published:** 2017-02-01

**Authors:** Trevor Gibbs

**Affiliations:** 1 AMEE

This review has been migrated. The reviewer awarded 2 stars out of 5 As someone who, like the author, is passionate about medical education, I often begin whatever teaching activity I am doing with the something around " medical education is about investing in the future". Wherever I am in the world, wherever my family and friends are, I want them to feel medically safe and assured that whoever looks after them is providing the best of care. In our all-to -busy world, care is frequently delegated down to the most junior of doctors , often on the spurious basis that there is someone else, more senior around somewhere if need be - that is where I believe the trust lies. If as a senior doctor I cannot be at all places at the same time, I need to trust my junior colleagues to handle my patient in the safest manner they can, I need to trust them. That trust has various levels, as described so well in the various papers on the subject, including those written by Olle ten Cate, and that trust-worthiness includes knowing what they can and what not they cannot do and when to shout for help. I feel that that trust also runs parallel with confidence. Although I am not converse with the paper by Skinner and Clemence, I am not so sure that in fact that high level of trust in the medical profession still exists in our patients, especially when there are so many stories of medical mishaps floating around the press and various social media - we need to regain that trust..Trust is what it means, raising that bar of assessment so that we can confidently say, yes, I am happy to let that student / graduate/ trainee treat my patient, without supervision and yes I would be happy for them to look after me and my family. I would agree with the author that lack of feedback, bullying and harassment in the work place are important issues that we must not let fall off the table, but isn't lack of preparedness all about trust and trustworthiness.

Reviewer Expertise:

NA

No decision status is available
